# Genetic determinants that permit growth without the core septin Cdc12 in *Cryptococcus neoformans*

**DOI:** 10.1093/g3journal/jkag240

**Published:** 2026-08-29

**Authors:** Piotr R Stempinski, Hannah S Akahoho, Rooksana E Noorai, Vijay Shankar, Lukasz Kozubowski

**Affiliations:** Department of Genetics and Biochemistry, Eukaryotic Pathogens Innovation Center, Clemson University, Clemson, SC 29634, United States; Department of Genetics and Biochemistry, Eukaryotic Pathogens Innovation Center, Clemson University, Clemson, SC 29634, United States; Clemson University Genomics and Bioinformatics Facility, Clemson, SC 29634, United States; Clemson University Institute for Human Genetics, Greenwood, SC 29646, United States; Department of Genetics and Biochemistry, Eukaryotic Pathogens Innovation Center, Clemson University, Clemson, SC 29634, United States

**Keywords:** fungi, *Cryptococcus*, septins, septin complex, transcriptional reprogramming, fungal morphology, cytokinesis

## Abstract

Septins are conserved cytoskeletal GTPases that regulate morphogenesis, cytokinesis, and cell-surface organization in fungi. In most model and pathogenic yeasts that proliferate by budding, including *Saccharomyces cerevisiae* and *Candida albicans*, core septin subunits such as Cdc3 and Cdc12 are essential and their loss results in lethal cytokinetic defects. In *Cryptococcus neoformans*, by contrast, deletion of *CDC12* or *CDC3* yields viable cells that grow normally under permissive conditions, raising the question of how this pathogen maintains cell integrity without a functional septin complex. To address this, we compared the transcriptomes of wild-type cells and a *cdc12*Δ mutant. The *cdc12*Δ strain showed a focused set of transcriptional changes, with induction of genes involved in cell wall remodeling, stress-dependent morphogenetic programs, and pathways that promote fitness under compromised cytoskeletal conditions. Genetic analysis of the most highly induced genes uncovered factors that contribute to proliferation in the absence of Cdc12, including a previously uncharacterized aspartic-type endopeptidase that exhibits a genetic interaction with the loss of septin function. These findings outline the compensatory circuitry that permits septin-independent growth in *C. neoformans* and help explain why this pathogen can tolerate the absence of the septin complex. This intrinsic plasticity highlights *C. neoformans* as a powerful comparative model for studying the evolution and function of septins.

## Introduction

*Cryptococcus neoformans* is an opportunistic yeast that causes life-threatening infections, particularly in individuals with weakened immune systems, such as patients with advanced HIV/AIDS, organ transplant recipients, and those receiving immunosuppressive therapies (Lui et al. 2006; Kwon-Chung et al. 2014; Perfect and Bicanic 2015; Santos-Gandelman and Machado-Silva 2019). Globally, *C. neoformans* is responsible for an estimated 194,000 cases of cryptococcal meningitis annually, leading to over 147,000 deaths, with the highest impact in sub-Saharan Africa (Lui et al. 2006; Kwon-Chung et al. 2014; Kabir and Cunningham 2022; Rajasingham et al. 2022; Denning 2024). Beyond its medical relevance, *C. neoformans* has emerged as one of the most important model organisms for studying fungal pathogenesis, stress adaptation, and morphogenesis (May et al. 2016; Zaragoza 2019; Brown and Ballou 2024). Its well-annotated genome, large number of genetic and molecular tools, and the availability of comprehensive mutant libraries have made it a key system for dissecting mechanisms of thermal tolerance, capsule biosynthesis, cellular division, and other virulence-related processes (Casadevall et al. 2000; Kronstad et al. 2012; Wang et al. 2012; Boucher et al. 2025). *C. neoformans* is a basidiomycete fungus, evolutionarily distinct from the ascomycete model *Saccharomyces cerevisiae*, offering a valuable complementary perspective on fungal cell biology.

Septins are conserved cytoskeletal GTPases that play important roles in cell morphogenesis, cytokinesis, and cell surface organization (Weirich et al. 2008; Bridges and Gladfelter 2015). In yeast cells, septins assemble into hetero-oligomeric complexes that form filamentous structures at the mother–bud neck, serving as a scaffold for proteins involved in cell cycle regulation, membrane compartmentalization, and cell wall maintenance (Gladfelter 2001; Douglas et al. 2005; Weirich et al. 2008; Varela Salgado and Piatti 2024). In many fungi, disruption of core septins leads to profound defects in cell polarity, hyphal morphogenesis, aberrant septation, and growth arrest, underscoring their importance in maintaining cellular organization (Longtine et al. 1996; Oh and Bi, 2011; Dagdas et al. 2012; Li et al. 2012; Dulal et al. 2020). The septins Cdc3 and Cdc12 are essential components of the septin complex in *S. cerevisiae* and *C. albicans*, and their loss leads to lethal defects in cytokinesis (Hartwell et al. 1974; Flescher et al. 1993; Warenda and Konopka 2002; McMurray et al. 2011). In contrast, cryptococcal cells lacking either Cdc3 or Cdc12 orthologs remain viable and proliferate relatively normally at optimal (nonstress) conditions (Kozubowski and Heitman 2010). In our previous work, we reported that among approximately 80 temperature-sensitive single-gene deletion mutants, the strain lacking the septin Cdc12 displayed the lowest maximal temperature (*T*_max_) at which cells remained viable and capable of proliferation (Stempinski et al. 2021). This variability highlights an important biological principle: the septin complex is both highly conserved and functionally flexible, and its structure and role can be modulated by organism-specific compensatory mechanisms. Given that other septins fail to assemble into a complex in the absence of Cdc12 (Kozubowski and Heitman 2010), it is likely that the functional septin complex in *C. neoformans* is expendable for cellular maintenance and integrity under optimal environmental conditions but becomes essential for survival at elevated, mammalian host-relevant temperatures (Kozubowski and Heitman 2010).

We hypothesized 2 nonexclusive scenarios that account for the viability of *C. neoformans* cells lacking Cdc12 under nonstress conditions. First, it is possible that septins are involved in stress response pathways. Second, it is plausible that the mutant that lacks Cdc12 has adapted to the absence of Cdc12 by transcriptional reprogramming such that the selected cells (dominant marker selection) survive at nonstress conditions. To address the question of what mechanisms account for the viability of *C. neoformans* cells lacking the core septin Cdc12 at permissive temperatures, we performed RNA sequencing of WT and the *cdc12*Δ mutant. Our results reveal a number of cell wall integrity and stress response-related genes as upregulated in the absence of Cdc12, suggesting a mechanism responsible for septin-independent proliferation. Notably, this study has identified a novel gene, a putative aspartic-type endopeptidase whose function is essential for compensating for the loss of septin function in *C. neoformans* cells. Morphological analysis further demonstrates that this gene positively contributes to fitness not only in the *cdc12*Δ background but also in the *cdc3*Δ mutant, supporting a broader role in buffering defects in septin function.

## Materials and methods

### Strains and media

The wild-type H99 strain and the congenic *cdc12*Δ mutant strain of *C. neoformans* were used in the RNA sequencing experiment (Kozubowski and Heitman 2010). Six deletion mutant strains were derived from the collection of single-gene deletion mutants of *C. neoformans*, and the absence of each deleted gene was confirmed by PCR (Liu et al. 2008). Frozen cell stocks were kept at—80 °C and streaked onto YPD medium (1% yeast extract, 2% peptone, 2% agar, and 2% dextrose) and incubated at optimal room temperature (∼24 °C). Fluconazole (Alfa Aesar, Cat. No J62015-03) was stored as a 50 mg/mL DMSO stock. Congo red was obtained from Sigma (Cat. No C6277).

### DNA isolation and PCR confirmation of the mutant strains

The DNA of tested strains was isolated from the overnight YPD cultures using the MasterPure Yeast DNA Purification Kit (Lucigen). Each strain was screened by PCR for the presence of the *CDC12* (CNAG_01740) gene, CNAG_05305 gene, and *CDC3* (CNAG_05925) gene as a positive control using the primers presented in Supplementary Table 1. The results of the PCR are presented in Supplementary Fig. 1.

### Cryptococcus neoformans RNA extraction

Cell cultures were grown overnight (18 h) at ∼24 °C in 25 ml of YPD medium. Five ml of overnight cultures were refreshed and incubated in 50 mL YPD for 3 to 4 h to reach an OD600 of ∼1.0. The cultures were incubated in 25 mL YPD at ∼24 °C for 1 h. After final incubations, cells were harvested by centrifugation at 3000*×g* for 5 min at 4 °C. RNA extraction was performed with Bio-Rad Aurum Total RNA Mini Kit (Cat # 732-6820). Cells were mixed with 750 µL lysis buffer and approximately 50 µl glass beads (Sigma G8772, 425 to 600 µm) and mechanically homogenized at 4 °C with Minibeadbeater (3450 RPM, Biospec Products Model 607) for eight 20-second beating cycles with 2 min rest in between. The supernatant with RNA was separated from the pellet by centrifugation (3000*×g* for 3 min) and treated according to the manufacturer's protocol. Each treatment and isolation was performed in 3 independent biological replicates.

### Transcriptomic analysis

Library preparation was completed using the Illumina TruSeq Stranded kit (Illumina, San Diego, CA, USA). Samples were sequenced to an average of 19.7 million reads per sample using an Illumina NextSeq 550 sequencing platform at 2 × 150 paired-end reads. Down-sampling was performed using seqtk v1.3-r106 (https://github.com/lh3/seqtk). Quality control was analyzed with FastQC v 0.11.6. Low-quality base sequences were trimmed using Trimmomatic v0.36. GSNAP v2018-07-04 software was used to count uniquely mapped reads that are aligned to reference genes. Statistical analysis for differential gene expression (DGE) was performed using edgeR v3.22.5. Analyzed genes were considered significantly differentially expressed if their false discovery rate (FDR) was less than 0.05. Supplementary Fig. 2 includes the principle component analysis (PCA) plot and the volcano plot associated with this analysis.

### Cryptococcus neoformans mating

Single-gene deletion mutant strains (MATα) (Liu et al. 2008), equipped with nourseothricin N-acetyltransferase (NAT) representing the group of genes with the highest upregulation in *C. neoformans* lacking Cdc12, were mated with a strain (a) carrying a *CDC12* deletion equipped with neomycin phosphotransferase II (NEO/G418) (Kozubowski and Heitman 2010). To induce mating, cultures of 2 different mating types (MATα and MATa) were grown overnight in YPD liquid at ∼24 °C, washed with PBS, mixed in a 1:1 ratio, and spotted onto MS mating media. Colonies were incubated in the dark at ∼24 °C for 10 to 12 d (Xue et al. 2007; Sun et al. 2019). For each strain, 32 spores, originating from at least 4 different basidia, were transferred onto YPD semisolid media with SporePlay Tetrad Dissection Microscope (Singer Instruments). To confirm the results of the mating and identify strains with double gene deletion, colonies were tested for growth on YPD media supplemented with a selection drug (NAT and/or G418), and the *cdc12*Δ *CNAG_05305*Δ double deletion mutants were confirmed by PCR (Supplementary Fig. 1).

To generate *cdc3*Δ *CNAG_05305*Δ double deletion strains, the *cdc3*Δ mutant (strain LK82, MATa, NAT) (Kozubowski and Heitman 2010) was mated with a *CNAG_05305*Δ deletion strain (MATα, NAT) (Liu et al. 2008). To induce mating, cultures of both strains were grown overnight in YPD liquid medium at ∼24 °C, washed with PBS, mixed in a 1:1 ratio, and spotted onto MS mating medium. Colonies were incubated in the dark at ∼24 °C for about 10 to 12 d (Xue et al. 2007; Sun et al. 2019). Since both parental strains carried a nourseothricin N-acetyltransferase (NAT) resistance marker, selection on antibiotic-supplemented media was not possible. Instead, bulk spore chains were collected from approximately 10 individual basidia and separated onto YPD semisolid medium using a SporePlay Tetrad Dissection Microscope (Singer Instruments). Individual spores were monitored until they germinated and grew into microcolonies, at which point the cells from each microcolony were transferred to separate locations on the YPD plate to form individual colonies. Once visible colonies grew, the colonies were picked and grown in liquid YPD medium and analyzed by PCR for the presence or absence of the *CDC3* and CNAG_05305 genes to identify wild-type, single deletion, and double deletion progeny (Supplementary Fig. 1). Strains were also tested phenotypically by DIC microscopy to assess cell morphology.

### Serial spot dilution assay

Overnight cryptococcal cell cultures were washed twice with PBS, adjusted to a final concentration of 3.3 × 10^4^ cells/μL, and used for a 10-fold serial dilution. 3 μL of cell cultures were spotted onto YPD media or YPD media supplemented with fluconazole (8 µg/mL) or Congo red (5 mg/mL). Plates were imaged after the cells were incubated at ∼24 °C for 72 h.

### Gene identification and GO analysis

Genes of *C. neoformans* identified in this study were analyzed using the FungiDB database (https://fungidb.org/) (Basenko et al. 2018). Functional analysis of the selected groups of genes was performed on the differentially expressed genes to identify their potential function using Gene Ontology (GO) for molecular functions and biological processes (Harris et al. 2004; Balakrishnan et al. 2013).

### Protein sequence analysis

Amino acid similarity analysis was performed using the protein-protein BLAST (https://blast.ncbi.nlm.nih.gov/Blast.cgi). Analysis of the secondary and tertiary structure of the protein encoded by the CNAG_05305 gene was performed using the AlphaFold 3 tool (Abramson et al. 2024). Computational protein localization prediction was analyzed using PSORT: Protein Subcellular Localization Prediction Tool (https://www.genscript.com/psort.html).

## Results

### Loss of septin Cdc12 induces cell wall remodeling and stress response pathways in *C. neoformans*

In *Saccharomyces cerevisiae*, deletion of the gene encoding septin Cdc12 is lethal due to its essential role in septin complex assembly and cytokinesis (Gladfelter 2001; Bridges and Gladfelter 2015). In contrast, while loss of Cdc12 in *C. neoformans* disrupts septin ring formation at the mother–bud neck, *cdc12*Δ cells remain viable at temperatures below ∼32 °C (Kozubowski and Heitman 2010; Stempinski et al. 2021). In this study, septin Cdc12 was selected as a core septin whose function is essential for septin complex assembly in fungi, making it a valuable target to analyze the consequences of septin network disruption in *C. neoformans*. We hypothesized that *C. neoformans* adapts to the absence of Cdc12 by altering gene expression patterns that support growth under optimal conditions in the absence of the septin complex. To test this hypothesis, we analyzed and compared transcriptomic profiles of the *cdc12*Δ mutant and wild-type (H99) strains grown in rich medium (YPD) at ∼24 °C. RNA sequencing revealed extensive transcriptional changes in the absence of Cdc12. A total of 134 genes were differentially expressed between the *cdc12*Δ mutant and wild type (FDR < 0.05). Strikingly, 131 genes were upregulated, while only 3 genes encoding proteins with unknown function were significantly downregulated (CNAG_06347, CNAG_04756, CNAG_00399), (Supplementary Table 2). All genes with logFC > 2 are listed in Table 1.

**Table 1.** A list of 23 genes with the highest level of upregulation (>2 logFC) in septin *cdc12*Δ in comparison to wt strain of *C. neoformans*.

Gene ID	logFC	Gene name	Product description	Protein size
CNAG_05305	5.4		Hypothetical protein	608 aa
CNAG_04891	4.2	Ril1	Ricin B lectin domain-containing protein	204 aa
CNAG_03833	3.6		Hypothetical protein	418 aa
CNAG_00587	3.3	Ril3	Ricin B lectin domain-containing protein	185 aa
CNAG_07399	3.3		Hypothetical protein	122 aa
CNAG_02339	3.1		DUF1275 domain protein	311 aa
CNAG_02381	3		DH domain-containing protein	479 aa
CNAG_05075	2.9		Solute carrier family 20	591 aa
CNAG_03650	2.8		Hypothetical protein	67 aa
CNAG_02544	2.6	SWI5/SAE3	DNA repair protein	102 aa
CNAG_05258	2.5	SMG1	Glucose–methanol–choline Oxidoreductase	588 aa
CNAG_03630	2.5		Hypothetical protein	246 aa
CNAG_01653	2.5	CIG1	Cytokine inducing-glycoprotein	281 aa
CNAG_07969	2.4		Hypothetical protein	309 aa
CNAG_00323	2.3		Kre9/Knh1-like domain-containing protein	167 aa
CNAG_00177	2.3		Hypothetical protein	530 aa
CNAG_00795	2.3	CFL1	Putative flocculin	309 aa
CNAG_04556	2.2	SEC62	Translocation protein	312 aa
CNAG_05458	2.2		Endo-1,3(4)-beta-glucanase	368 aa
CNAG_07877	2.2		Hypothetical protein	175 aa
CNAG_06715	2.1		Hypothetical protein	198 aa
CNAG_01994	2.1		SET domain-containing protein	1,006 aa
CNAG_06027	2.1		Aryl-alcohol dehydrogenase	400 aa

Among the upregulated genes, 55 (42%) were either previously characterized or could be inferred by sequence homology. Notably, several genes associated with cell wall biosynthesis and remodeling were strongly induced in the *cdc12*Δ mutant, including *CHS5* (CNAG_05818) and *CHS7* (CNAG_02217), encoding chitin synthases, *AGN1* (CNAG_07736), which encodes a glucan endo-1,3-alpha-glucosidase, and 3 glucanases (*CNAG_05458*, *CNAG_06336*, *EXG104*/*CNAG_02225*), as well as a glycosyl hydrolase, or as more recenlty classified, lytic polysaccharide monooxygenase (*CEL1*, CNAG_00601) (Banks et al. 2005; Reese et al. 2007; Donlin et al. 2014; Rodrigues et al. 2018; Mukaremera 2023; Probst et al. 2023). The upregulation of these genes suggests that reinforcement or remodeling of the cell wall may compensate for the loss of structural integrity caused by the absence of a functional septin scaffold.

In addition to genes associated with cell wall and surface architecture, we observed upregulation of 2 components of the iron acquisition system essential for cryptococcal virulence: the cytokine-inducing glycoprotein *CIG1* (CNAG_01653) and the ferric transporter *CFT1* (CNAG_06242) (Jung et al. 2008; Han et al. 2012; Cadieux et al. 2013; Garcia-Santamarina et al. 2017). Furthermore, 2 genes encoding a glutathione S-transferase (*CNAG_01874* and *CNAG_04110*) involved in glutathione metabolism and reactive oxygen species homeostasis were also significantly upregulated, suggesting activation of oxidative stress response pathways (Missall et al. 2005; Wangsanut and Pongpom 2022). Finally, *CFL1* (CNAG_00795), encoding a putative flocculin, showed one of the highest levels of induced expression (Wang et al. 2012). Cfl1 is known to be a hypha-specific adhesin and among the most strongly expressed genes during sexual development, suggesting activation of another mechanism supporting cellular morphology and cellular growth in the absence of the septin complex (Wang et al. 2012).

We next performed Gene Ontology (GO) term analysis to characterize the biological processes, molecular functions, and subcellular localization of the proteins encoded by genes differentially expressed in the *cdc12*Δ mutant strain (Fig. 1). The analysis of biological processes showed the presence of multiple genes involved in responses to stress, abiotic stimuli, and heat stress (Fig. 1a). Additional categories included genes involved in the oxidation–reduction process and carbohydrate metabolism. GO analysis of molecular function further highlighted that the dominant protein groups are engaged in oxidoreductase and hydrolase activities (Fig. 1b). Finally, GO analysis of cellular localization indicated that most proteins are integral membrane proteins and are localized to peripheral and extracellular regions of the cryptococcal cell (Fig. 1c).

**Fig. 1.**
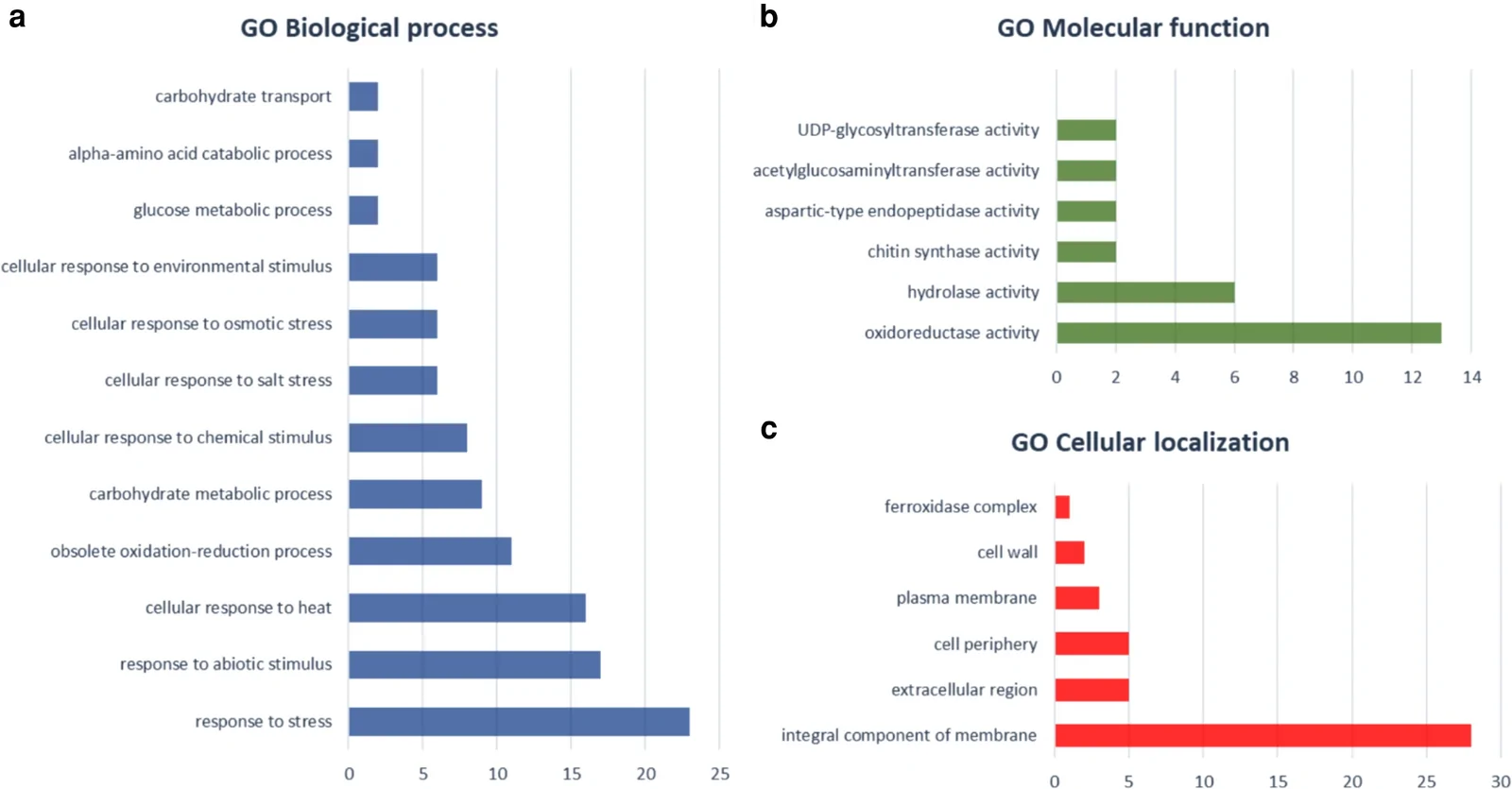
Bar graphs representing GO terms analysis for a) biological processes, b) Molecular function, and c) Cellular localization of *C. neoformans* genes upregulated in the *cdc12*Δ mutant. The *x*-axis represents the number of genes in each GO term.

### Phenotypic analysis of strains lacking genes that were highly upregulated in the absence of septin Cdc12

To explore the nature of the putative suppression of a potentially deleterious phenotype in the absence of the septin complex, we selected 6 mutant strains carrying deletions of the most upregulated genes in the *cdc12*Δ mutant strain. The protein sequences of all 6 highly upregulated candidates were analyzed using BLASTP (https://blast.ncbi.nlm.nih.gov) and FungiDB (https://fungidb.org/) to identify potential homologs in other fungal species. Interestingly, homologs of these genes were found only in the order Tremellales, which includes the genera *Cryptococcus* and *Tremella*. Serial dilutions of WT, *cdc12*Δ, and those 6 selected mutant strains were spotted onto YPD media supplemented with the cell wall-disrupting agent Congo red or a subinhibitory concentration of the plasma membrane-disrupting antifungal drug fluconazole and incubated at 30 °C or 37 °C (Fig. 2). Consistent with previous observations, incubation on solid YPD at 37 °C confirmed that *cdc12*Δ was extremely sensitive to elevated temperature, whereas all other deletion mutants and the WT strain grew well. Interestingly, deletion of gene CNAG_05305 resulted in a drastic growth defect on YPD supplemented with 8 µg/mL fluconazole, exceeding the previously reported sensitivity of *cdc12*Δ mutant cells (Kozubowski and Heitman 2010; Stempinski et al. 2021). This finding suggests that CNAG_05305 plays a role in plasma membrane homeostasis and/or stress response pathways that are critical for cell vitality in the absence of a functional septin complex. In contrast, supplementation with 5 µg/mL Congo red produced mild growth inhibition of the *cdc12*Δ strain and had no visible impact on the other mutants. These results suggest that neither of the 6 analyzed genes is essential under cell wall stress conditions. Furthermore, among the 6 genes, only CNAG_05305 was essential under conditions that compromised the plasma membrane.

**Fig. 2.**
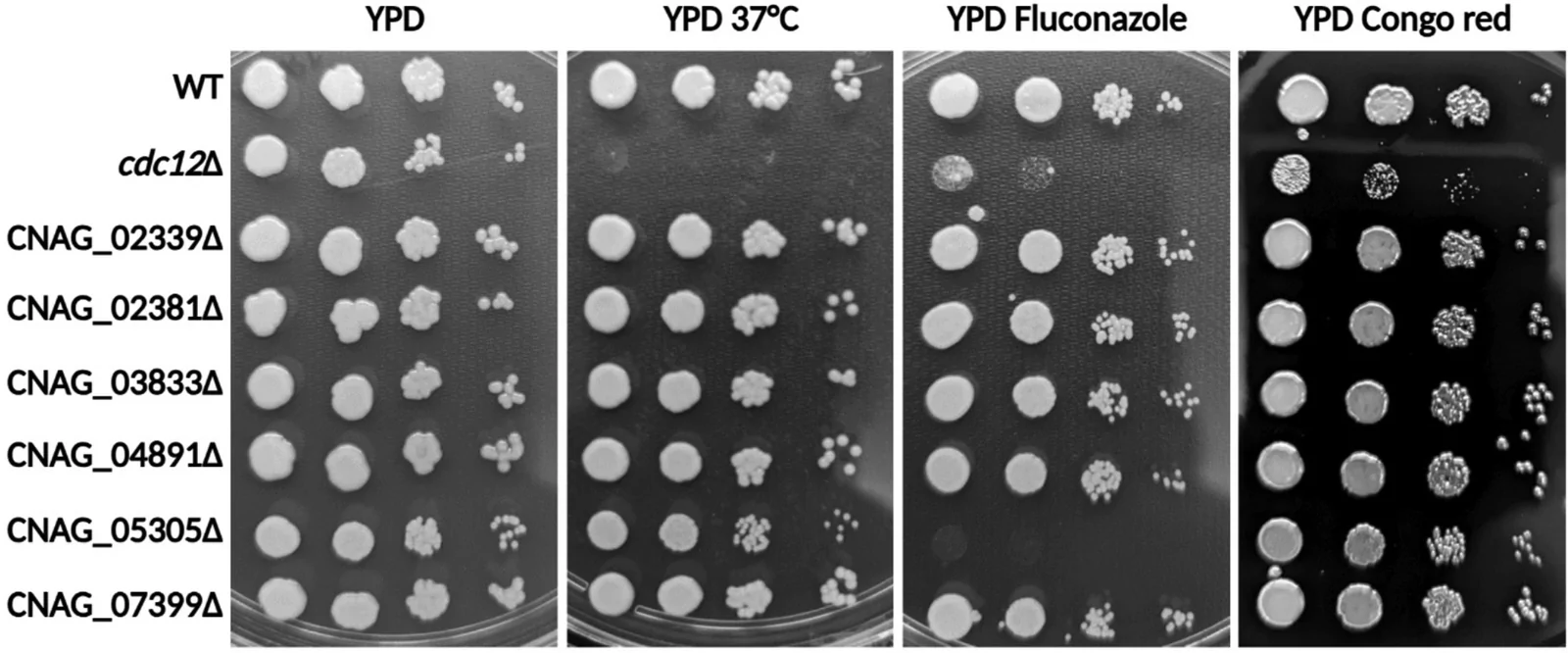
Spot assay of WT, *cdc12*Δ, and selected mutant strains. Threefold serial dilutions of each cryptococcal strain were spotted onto YPD media, YPD media with 8 µg/mL fluconazole, and YPD media with 5 µg/mL Congo red. Plates were incubated at 30 °C or at 37 °C as indicated.

### Genetic interaction between CNAG_05305 and the genes encoding septins Cdc12 and Cdc3 highlights compensatory mechanisms important for morphogenesis

The septin complex plays a vital role in regulating the cell cycle and cell wall synthesis in *S. cerevisiae* (Gladfelter 2001; Douglas et al. 2005; McMurray et al. 2011). *C. neoformans* cells lacking a functional septin complex display aberrant morphology of the hyphae, increased sensitivity to cell wall-damaging agents, and significantly decreased virulence (Kozubowski and Heitman 2010). Loss of septin function is predicted to produce pleiotropic morphological defects, including cytokinesis abnormalities. Interestingly, at temperatures below ∼32 °C, the *cdc12*Δ mutant maintains largely normal morphology, likely due to a supportive gene expression profile.

As high expression of a gene in the absence of Cdc12 suggests this gene is essential in the absence of this septin, we hypothesized that eliminating both *CDC12* and such a gene would lead to a synthetic lethal or synthetic sick phenotype in the double mutant. To confirm the supporting role of selected genes in the septin-deficient strain of *C. neoformans*, we performed mating between the *cdc12*Δ mutant and deletion mutants corresponding to the group of most strongly upregulated genes in the *cdc12*Δ background. Among all tested progeny, only the double deletion of *CDC12* and CNAG_05305 resulted in an apparent growth defect (Fig. 3). We observed occasional variation in colony size among progeny from several genetic crosses. These small-colony phenotypes were limited to individual progeny and likely reflect phenotypic variation during meiotic recombination or delayed spore germination. In contrast, the growth defect associated with the *cdc12*Δ *CNAG_05305*Δ genotype was consistently observed across multiple independent double mutant progeny.

**Fig. 3.**
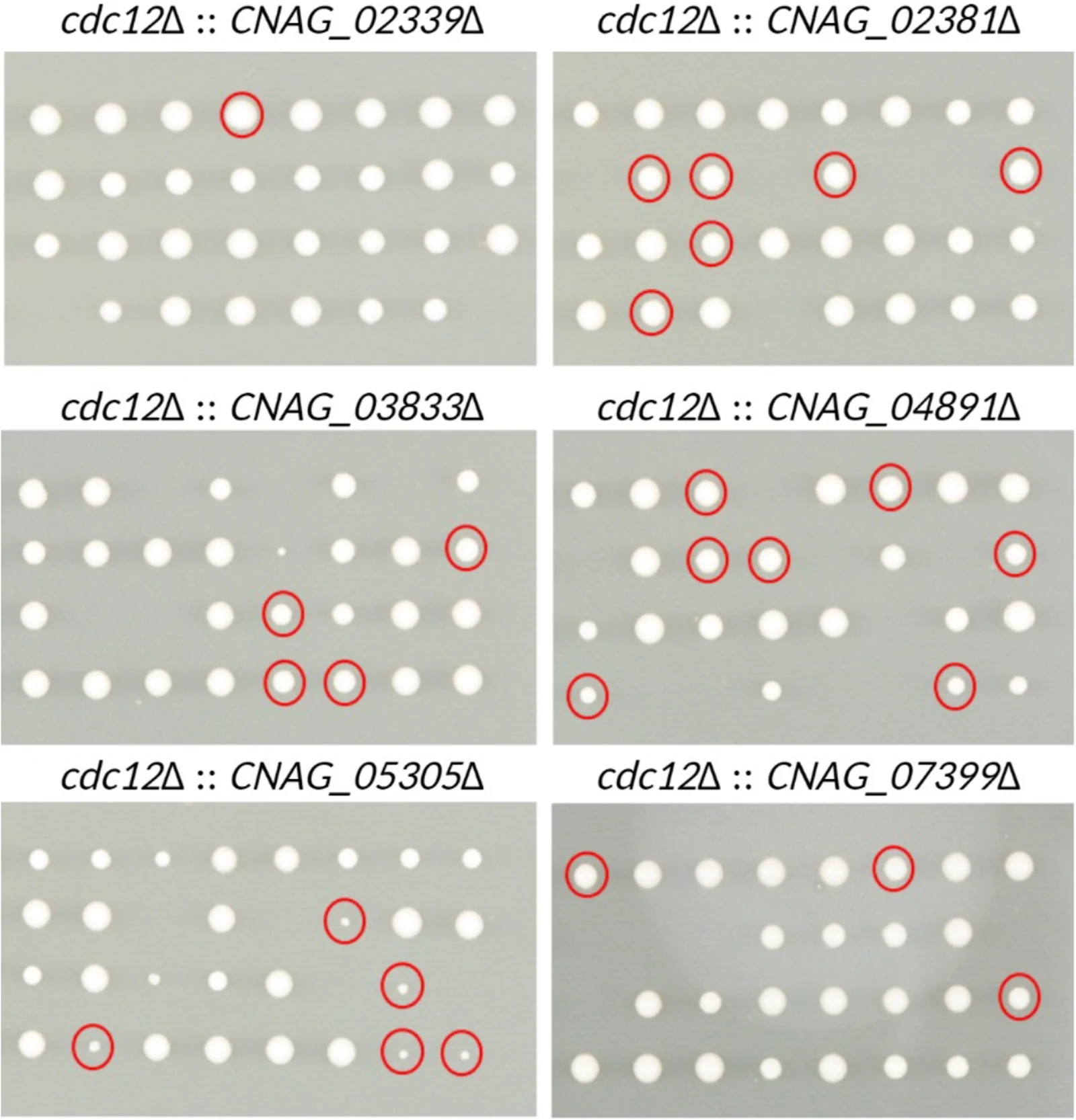
Qualitative growth analysis of single spore colonies. Spores were obtained by mating of the *cdc12*Δ mutant with deletion mutants of the genes with the highest upregulation in the *cdc12*Δ background. Spores were transferred onto YPD plates and incubated at ∼24 °C. Colonies surrounded by circles indicate confirmed colonies of double-deletion mutant strains.

Microscopical analysis confirmed previously published observations that most *cdc12*Δ cells grown in YPD at ∼24 °C resembled wild-type cryptococcal cells, with only a minor fraction of the population presenting elongated or misshapen forms (Fig. 4a). Similarly, the single deletion of CNAG_05305 resulted in predominantly normal cellular morphology, with some cells exhibiting a clustered phenotype. In contrast, 4 independent progenies representing the double-deletion mutant showed severe morphological defects across the entire cell population, suggesting an important role for CNAG_05305 in maintaining proper cell shape and integrity in the absence of Cdc12 (Fig. 4a).

**Fig. 4.**
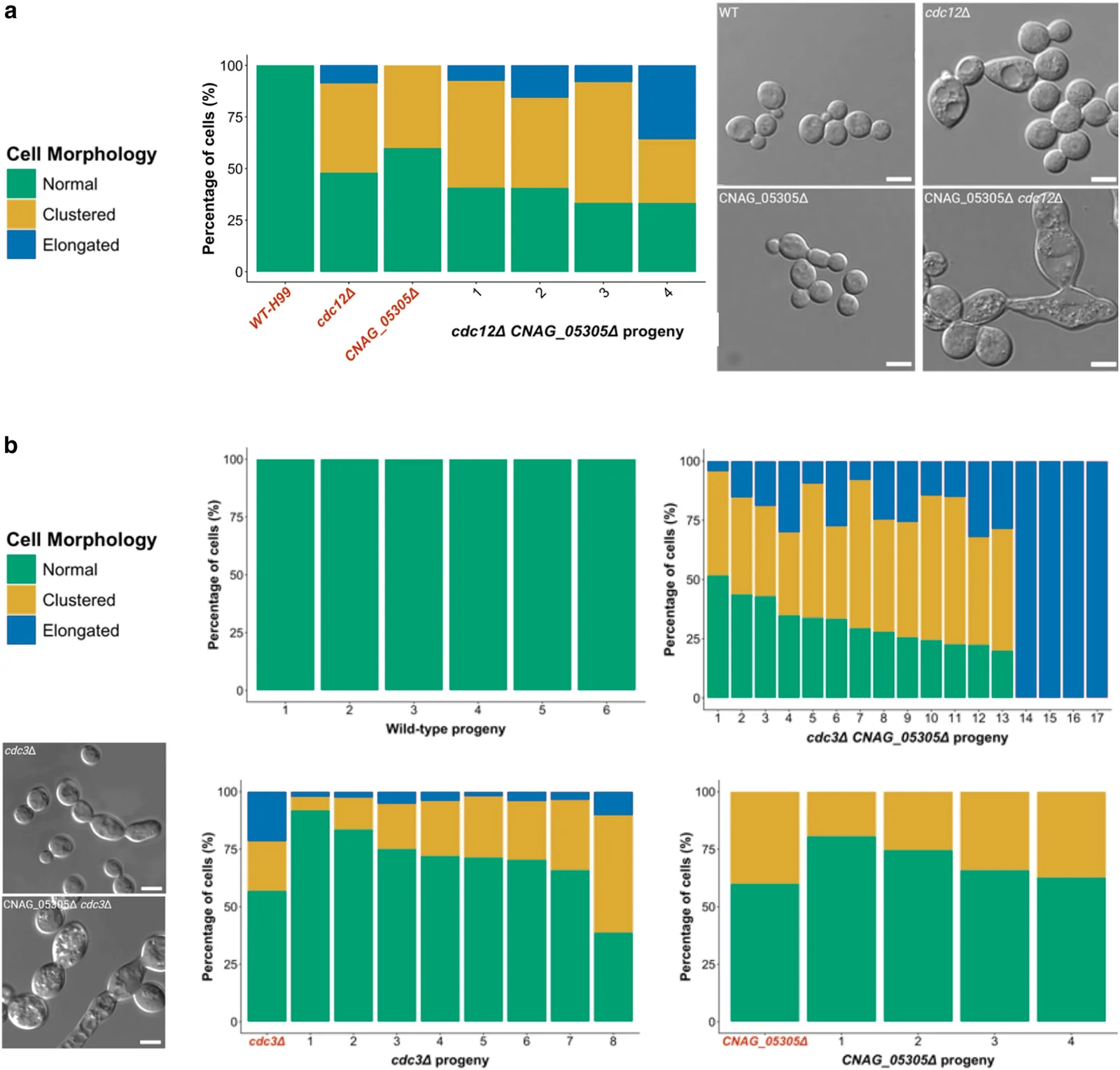
Cell morphology of the WT and deletion mutant strains. a) Percentage of normal, clustered, and elongated cells for WT (H99), the parental *cdc12*Δ strain (LK174), the parental CNAG_05305Δ strain and 4 independent *cdc12*Δ CNAG_05305Δ double deletion mutant progeny obtained by mating. WT, *cdc12*Δ, and CNAG_05305Δ reference strains are indicated in bold red italic on the *x*-axis; double deletion strains are numbered 1 to 4 and ordered from highest to lowest percentage of morphologically “normal” cells. All double deletion strains were confirmed by PCR genotyping (lanes 4 to 7, top panel, in Supplementary Fig. 1). b) Percentage of normal, clustered, and elongated cells for 2 parental strains (the *cdc3*Δ mutant, LK82, and the CNAG_05305Δ mutant) and 35 progeny derived from the mating, including 6 wild-type progeny, 17 *cdc3*Δ CNAG_05305Δ double deletion mutant progeny, 8 *cdc3*Δ progeny, and 4 CNAG_05305Δ mutant progeny (ordered from highest to lowest percentage of normal cells). All genotypes were confirmed by PCR (in Supplementary Fig. 1, lanes 4, 6, 8, 9, 10, 11, 12, 13, 14, 16, 17, 20, 24, 25, 26, 29, and 35 are double mutants; the 8 *cdc3*Δ progeny strains and 4 CNAG_05305Δ progeny strains are lanes 2, 5, 7, 15, 21, 22, 27, and 34, and lanes 18, 23, 28, and 38 respectively). For A and B, an average of 100 cells was scored per strain based on DIC microscopy images. DIC images included in A and B illustrate representative cells for each genotype. Bars represent 5 microns.

The observed synthetic interaction between deletion of CNAG_05305 and *CDC12* has likely resulted from the absence of the septin complex, as in the mutant lacking Cdc12, the septin complex fails to assemble at the mother-bud neck (Kozubowski and Heitman 2010). Alternatively, the genetic interaction may be specific to *CDC12*. To differentiate between these possibilities, we analyzed the morphology of the progeny obtained from a cross between a strain lacking CNAG_05305 and a mutant that lacks Cdc3, another septin that is critical for septin complex formation (Kozubowski and Heitman 2010). Reassuringly, genotypically wild-type progeny were morphologically normal, and the single deletion mutants lacking either CNAG_05305 or the *CDC3* gene showed phenotypes similar to those of the parental strains, although the percentage of morphologically abnormal cells varied among each progeny class, suggesting segregation of other genes has influenced the penetrance of the morphological defect (Fig. 4b). Importantly, the double mutant lacking CNAG_05305 and *CDC3* revealed a potential synthetic interaction between those genes, as a great portion of the analyzed population exhibited the most severe morphological abnormalities indicative of pleiotropic defects including inability to complete cytokinesis and/or cell separation (Fig. 4b). Thus, our data suggest CNAG_05305 has a critical role in maintaining cell integrity in the absence of the septin complex.

### Structural and *in silico* functional analysis of CNAG_05305 predicts its product as a putative aspartic peptidase with a CCHC-type zinc finger motif sequence

Initial *in silico* analysis of the cryptococcal protein encoded by CNAG_05305 using the AlphaFold 3 Protein Structure Database identified it as a protein of unknown function with a predicted molecular mass of approximately 68.1 kDa and a tertiary structure comprising 3 distinct protein domains (Fig. 5, a and b). The amino acid sequence analysis using InterProScan indicated the presence of an aspartic-type endopeptidase domain and a CCHC-type zinc finger motif domain, which is typically associated with nucleic acid binding (Fig. 5c) (Benhalevy et al. 2017; Wang et al. 2021). Detailed analysis of the indicated CCHC-type zinc finger-like motif revealed the existence of a 13-residue domain with the CX_2_CX_3_HX_4_C consensus sequence (CFTCGAYHWSDQC). Further evaluation of the zinc finger-like domain in the protein sequences from homologous proteins in other, closely related fungal species confirmed the highly conserved character of this domain (Fig. 5d and Supplementary File 1). Subcellular localization analysis using the WoLF PSORT tool predicted that the protein encoded by CNAG_05305 is most likely localized to the nucleus or the cytoplasm, further supporting a regulatory or processing role for this protein.

**Fig. 5.**
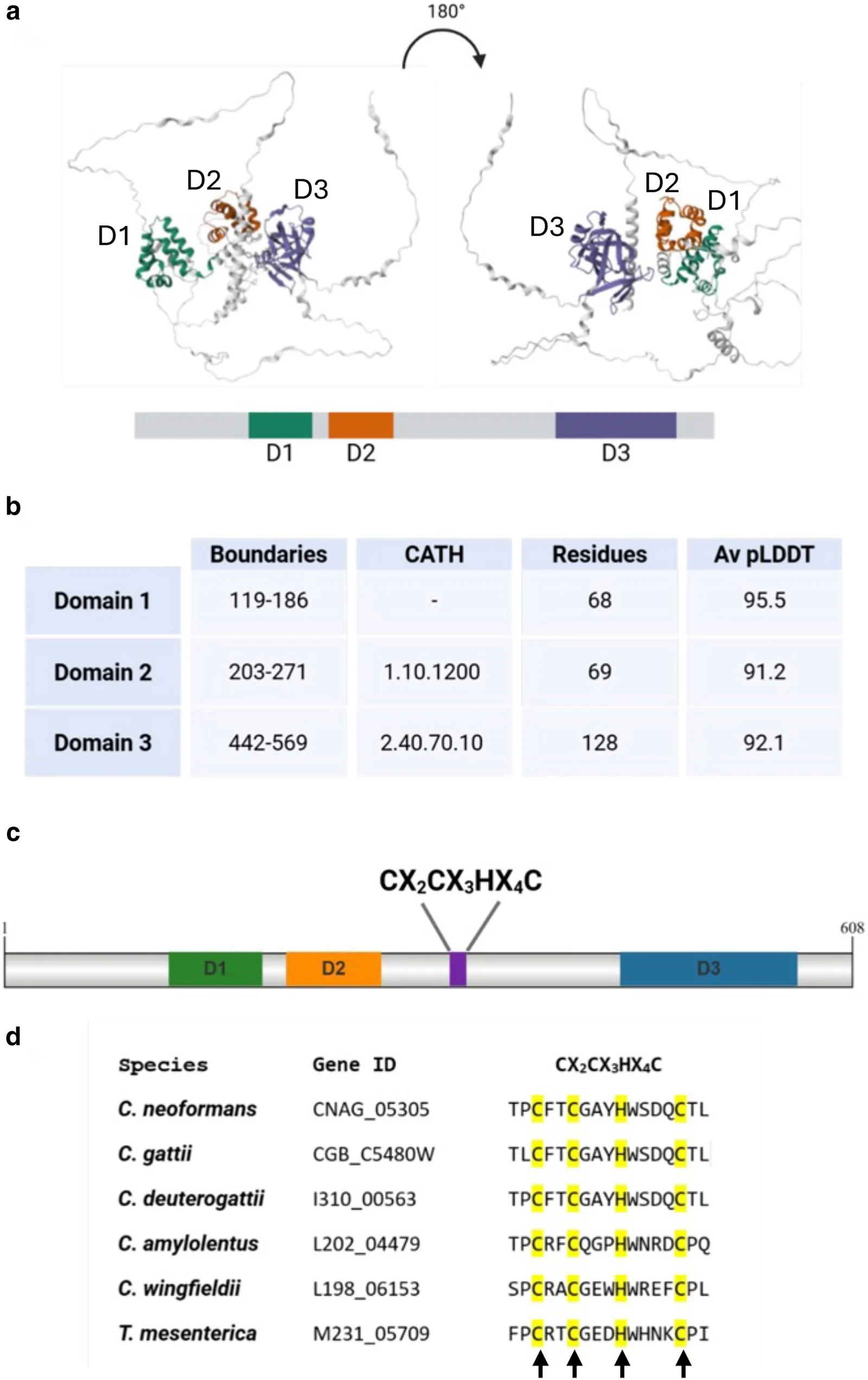
Predicted structure and domain organization of the CNAG_05305 protein product, a putative aspartic peptidase. a) Predicted three-dimensional structure of the CNAG_05305-encoded protein generated using AlphaFold3. Two orientations of the model, rotated 180° along the *x*-axis, are shown to illustrate the overall protein architecture. The 3 predicted structural domains identified by AlphaFold3 are labeled as domains D1, D2, and D3, with the aspartic peptidase DDI1-type domain labeled as D3. b) Summary table of the structural features of the CNAG_05305-encoded protein, indicating the boundaries of each domain, corresponding CATH (Class, Architecture, Topology, and Homologous Superfamily) classification, number of residues, and average predicted local distance difference test (pLDDT) score. c) Visualization of the putative zinc finger motif, represented by a 13-residue region containing the conserved CX_2_CX_3_HX_4_C sequence within the protein. d) Multiple sequence alignment of the CX_2_CX_3_HX_4_C zinc finger region from several *Cryptococcus* and *Tremella* species. Conserved cysteine and histidine residues are highlighted in yellow and marked by arrows.

## Discussion

Comparison of the septin complex biology in *S. cerevisiae* and other fungi emphasizes its conserved and versatile functions. In *S. cerevisiae*, Cdc12 is essential for septin complex assembly, and an intact septin complex plays a critical role in cytokinesis (Bridges et al. 2014; Bridges and Gladfelter 2015). In contrast, *C. neoformans* cells can tolerate the absence of the septin complex under optimal growth conditions, reflecting species-specific differences in septin network robustness and adaptive capacity. Similar temperature-sensitive phenotypes have been reported in other pathogenic fungi, suggesting that septin-dependent processes are particularly critical under host-relevant stress conditions.

The main goal of this study was to investigate the putative supporting molecular mechanisms that allow *C. neoformans* to proliferate in the absence of Cdc12 under “optimal” growth conditions. Transcriptomic analysis suggests that the *cdc12*Δ mutant requires a major transcriptional adaptation, characterized by the increased expression of multiple genes involved in cell wall biosynthesis and integrity, oxidative stress response, and nutrient acquisition. This adaptation likely enables *C. neoformans* cells to maintain cellular integrity and viability in optimal environmental conditions, despite the loss of a key septin component that is essential in most other fungal species. Identification of genes encoding membrane-associated proteins and extracellular components suggests that septin deficiency impairs cell surface integrity.

The fact that 131 genes were upregulated in the absence of Cdc12 suggests a complex compensatory program to sustain growth in its absence. It would therefore be expected that none of the upregulated genes is essential in the *cdc12*Δ mutant. Rather, several genes collectively contribute to viability in the absence of Cdc12. However, elimination of the single most upregulated gene, CNAG_05305, led to a synthetic sick/lethal phenotype with both *cdc12*Δ and *cdc3*Δ. The severity of morphological abnormalities resulting from the CNAG_05305Δ *cdc12*Δ and the CNAG_05305Δ *cdc3*Δ double deletions varied among received mutant progeny, suggesting recombination of other genes has influenced the penetrance of this phenotype. Some of the spores collected from either of the crosses have not formed colonies (Fig. 3 and data not shown), suggesting a synthetic lethal phenotype, although this could not be confirmed, given difficulty in performing reliable segregation analysis in this species. Nonetheless, the synthetic sick phenotype resulting from deleting CNAG_05305 and either *CDC12* or *CDC3* points to an important compensatory role of CNAG_05305 in the absence of the septin complex.

CNAG_05305 may support *C. neoformans* cells lacking the septin complex via several potential mechanisms. The analysis of protein sequence revealed that CNAG_05305 encodes a putative aspartic-type endopeptidase with a CCHC-type zinc finger motif, features often associated with RNA metabolism and general transcriptional regulation (Benhalevy et al. 2017; Wang et al. 2021). The predicted cytoplasmic or nuclear localization of this protein suggests that its function may involve regulating stress-related transcription or mRNA dynamics important for indirectly reinforcing the plasma membrane or cell wall under stress. The putative role of CNAG_05305 in plasma membrane homeostasis is supported by our results showing hypersensitivity of cells lacking this gene toward treatment with fluconazole. The abnormal phenotype observed in the double-deletion mutants lacking CNAG_05305 and *CDC12* or *CDC3* supports the idea that the septins and the product of CNAG_05305 operate in parallel, partially overlapping pathways that together maintain cellular organization and morphogenesis. Importantly, while these genetic interactions demonstrate that CNAG_05305 contributes to cellular fitness when septin function is compromised, they do not establish that the protein directly compensates for loss of the septin complex. Rather, our findings identify CNAG_05305 as a component of the adaptive response that becomes critical under conditions of septin dysfunction. This observation is further supported by the fact that in the *cdc12*Δ strain, CNAG_05305, present only in closely related fungal species, showed the highest level of upregulation among all detected genes (>5 logFC).

Together, our findings reveal that *C. neoformans* adapts to the absence of a key septin through unique transcriptional reprogramming and activation of genes, such as CNAG_05305, which are uniquely present in the genomes of fungi that belong to the order Tremellales. This increased adaptive capacity may explain why elimination of the septin complex in *C. neoformans* is not lethal in contrast to *S. cerevisiae* or *C. albicans* (Flescher et al. 1993; Frazier et al. 1998; Warenda and Konopka 2002). Future studies will shed more light on the specific role of CNAG_05305 in *C. neoformans* morphogenesis and on the mechanisms underlying its contribution to stress response and antifungal resistance.

## Supplementary Material

jkag240_Supplementary_Data

## Data Availability

Strains are available upon request. Supplementary files contain Supplementary Fig. 1, Supplementary Fig. 2, Supplementary File 1, Supplementary Table 1, and Supplementary Table 2. Gene expression data are available at GEO repository with the accession number: GSE315847. Supplemental material available at G3 online.
