# Supplementary material for: Genetic determinants that permit growth without the core septin Cdc12 in Cryptococcus neoformans
Source: G3 (Bethesda). 2026 Aug 29;16(11):jkag240. doi: 10.1093/g3journal/jkag240 (PMC13732481; doi:10.1093/g3journal/jkag240)
Supplement: Supplementary file 1 [file jkag240_supplementary_data.zip › Supplementary_File_1_G3-2026-407083.pdf]

***Cryptococcus neoformans* H99**

>XP_012048840.1 hypothetical protein CNAG_05305 [Cryptococcus neoformans H99]

MNNRLATHLQHVDTIYQQRFNELHDLFQATHGATLRHSTPLHQADPSLDTSSSTSPTPPLRPTSPPTCPP

TQPPTIPLDPPSSTSVIGKNDSVLSTSQPTRLKASDLPKFYGRDDDDIVYWVQKISSIKRGSQATDSDIL

RLLPSLLRDNAFKYYARLPEKEHTSLTTWAAWSKELQDRFLPPNSLDDLKEKCIRRYLGPNETFANYYED

KVYLQQFLFPANTEDSLLVRDILGGIPATLRAQLQTGVTPTMSLREFRLHVLQIEPRFHPQLFPTSHRHP

ADRTRTQLNNHSNTSFANSSIARSSRFLPGNRPEKPSTPCFTCGAYHWSDQCTLKKRQQQQYNNSRPSFT

TNNPATSTSSDGRSSFPSWARPPSNTNSPNYKPPANSVNSIANKDGYRFDSPKPSIHTLCHSTFPRPSAH

LPTHALPQLQLHRTHLNKTPAYATVKFNSNNPSATTHRAVIDTGASLSAISQEYANKHLSHLPRHPFTNF

QLNGIGKSSSAGYHLVDIHFPTKEGYHIVIPTALFVIDNISVDLILGLDFLLPHSAKIDLRDGILSFPRH

SGTILLSCLSSALPFPPIPLPRPPRRFIAKSSRVQTMLFPDPVAERTI

***Cryptococcus gattii* WM276**

>XP_003192864.1 Hypothetical Protein CGB_C5480W [Cryptococcus gattii WM276]

MSTSPSTRPRALSVGADARSQDSIFPPPSIVSPRRMSITPQPKPLAHEIAHHYEVEIENRVTTCLKRIEE

VHQQRLNELNTLMQSMSDMMPTVSSHSIPLDHIVSSPNTSLPSLPIPPVPLRHTLPSQPTVTSTPTQTSA

QPTRLKASDLPKFYGRDDDDIVEWVQKVGSLKRVFEATDRDILRLLPSLLHELQDRFLPPNRLDDLIYRC

IRRYLGQNEKFVTYYEDKVYLQQFLFPANTEDFLLIRDILSGILSTLRAQLQTAVTPNMSLCDFRCHVLQ

IEPGFRPHLFPSSHRHPADRARHQRNNHPQRSSDTSTSARPFRHTPGTRPDKPCTLCFTCGAYHWSDQCT

LKRQQHQQYGARPPPATNNPGSSTSSTSRRFFLSWARPASNTTSHSYQPLSNSVNSIQPKDNNRFNSPKP

SINTLLHSHSPAPKPLHALPPLQLQQKHLNMTPAFANIKFNSNHPSALTHRAVIDTAPGHWQILLSRISP

G

***Cryptococcus deuterogattii* CA1014**

>KIR75865.1 hypothetical protein I310_00563 [Cryptococcus deuterogattii CA1014]

MYITMRHPVPTPCFTCGAYHWSDQCTLKQQQQQQHNATWPSSNANNPATSTSLNSCSSFPSWACPSPNT
N

SSNYKPPANLVNSIANKDGYCFNSPKPSVHTLLHSTPTCSSTHSPVHAIPQLQLQQTHLNKTPAFATVKF

NSNHPFASTHHAIINTGASLSVIRQEYAQKHLSHLLCHPFKNFQLNGIGQSSSTGYHLIDIHFLTKEGYN

TIILTALFIINNISVNLILGLDFLLPHHAKIDLQDGILSFPCHPGTILLSCQSGPLIPPHLPPLPSPIPC

PFVPSIHVKEAFTILPVHQAHIETTIDVLPHTPQYLVSPTSTGKYLHVACCVGSSTASKHFVLVLNTGDR

PIPVSGGHLLGYPLPLHTACPTPAVHTINTVAIPETDSDAEPFPITDLKINPDLTPEQSEALHQVLMEQC

HIFGFGSQQLGSTNLATMNVDTGNHPPISTPPYRISPQGHATIDEKLTELLNQGIIEEADSPWASPAILV

***Cryptococcus amylolentus* CBS 6039**

>XP_018994010.1 hypothetical protein L202_04479 [Cryptococcus amylolentus CBS 6039]

MAQPATQPSRRNLRSQGDALLDGPADVVTNQPQPTPTLPSGPSLQSEVDIRPSNFQPPPSIDDTPTSEHH

ITTHQDLVHGPAPTVGYLMHQNQSLLSSLAEMRDEMRSLRLSQSRDTTPVSPPSRPHAPKLKHEMFPKFR

GQDNEDVDTWVTSVTAIFEFSGAADTDLLLLLPALLQGSAQRWFTNLEPERRRSLCTWSQWSEAIRSVYL

GANHASRLRNKCIFRRLKTTDGLSKDIYPAGTPDVDRIDDIINGLPEHMRKLARIDAQLHPNLDDFRRAL

IDGESSMRPELKQNRRRQDKAPPTSQNKHQQRDDKNSSSTLSSPRTPCRFCQGPHWNRDCPQQATAPPRP

TQTMAQPNPSPSTAPTYGNFRLASRPDSDRPLGFRPAPASANGTPLGSRSSSVETTSGPIKSITVSGAVS

LGSTVAELGTETVGGVDTWNARGARERGGDSRNVGRGGSLSLGIRDSRSLVGGGSLSLGIGVSRSLSRGR

RSGPYEHGVSTIWLYTCHCTANSSGKKVESPRKPPQTHEQQTTLSCFYGNESW

***Cryptococcus wingfieldii* CBS 7118**

>XP_019029938.1 hypothetical protein L198_06153 [Cryptococcus wingfieldii CBS 7118]

MPPATRSGPSSAPVPSDADTPTSSLTSLADPVETGGPSQAQSQADPSSEVSNLGDAVVPQTLPAEDGTAT

DQSQVASGSRATDHPTDLPARGTSLAPSETPSSWHSDLQVLSQNQALLTRLLHTMHEQNILRAQSLPTSQ

TPPPPDNPSSQPSFERRRRLRPADLTKFGGSDTEDVDMWLEKLTAALEHADYPESELLSNLPFLLEGKAL

DWFTDLGPVRRDYQTWDEWRVVFKNAFRIPDFEGVMRRKCIARRLQPFESFADYFDDKRRLQRWVYPVGT

SSKDLITDIVEGIPLVMRALIKASTPPGASLDDFRRIMLDLQPSLRSQFPTPNDKPPRVHNDDQQDRSRT

APSRQSPQTRAAAPPSPCRACGEWHWREFCPLNSPRPFNTPPSDSYGRSQNGFDDHQRHDSQSRTFAGSG

SNGISRPSDNGYQDQQDTRPFQSHSPGNV

Tremella mesenterica

>RXK37002.1 hypothetical protein M231_05709 [Tremella mesenterica]

MSTPNIPSTDQGLANPGTNTHTQVTDDHIRTLCSQLLNHHNTNIDNQIVQLQNAIDHLESTHAQEIDDLL
SQLNDLQEKCDNLQHQLDTTTNISVPTTNHSTVPLVNSTSHIKLKVTELPKFTANSTSESVDDWVNKITA
ILQQSGAPDTEITAKIPLLLQFAALDWFNTLSNIDRAKCKTWADWSTLFKSTFRAANYSAKLLALARDRH
LLPEESVTTYFWQKVKLLRDVHGSLITNQVLVNEILAGLPTSMSTFLNVNPNIELTEFHRLLVDKEDGLR
TLGFTPNFSTNLDQDQDTTNIFDNNYDSEEDVLDNYANNTNTIPTAPPFPCRTCGEDHWHNKCPIRNGVY
HDNNTIDHSNHDNADTYSHHDPAAPDNNSHNWGHHQNTCTSNKTIYKDNPNLQPITKPRQWVPLNILFCL
ETSSDTQLSGSQTPQAHQQAIGLVRKMGSFFSHRRASSFPLPWSSSQDESIFSTSTYEPPSYQNDNGSFN
IIQSKLFSRTRDHGTGESTMWGKIKSWFRLNSSVSYSNPTIPPELYVTLAESLTRLQLHKTLLNFSLSSK
SHYRLISPVLYREAILPLNDNVVDKLGDFYLHYKIFPQSRDSLIDPDFSFLTWDPNYLASSTSARLAWRC
QWARTVRIRTSPQTLQSLSMTSIVQTLSKIELCLFPRLTKLSLRRSASRKSATLLRLLALKSEIDHLLLF
LGSSHLDIHFPWFRDYLTLHDPVHIDRMVVRVDIKKNKWYFYPVKLSPTTGDDRNVSKDDFERQFDAKKS
DRDTLPRFHILSSPPKDRRRSTVASLNHYLKENTNEAGNFAKILTLGSVDLDPCIVFDPDKQKAIICRKI
RIVKKEVGQ

	**$CX_2CX_3HX_4C$**
C. neoformans	TPCFTCGAYHWSDQCTL
C. gattii	TLCFTCGAYHWSDQCTL
C. deuterogattii	TPCFTCGAYHWSDQCTL
C. amylolentus	TPCRFCQGPHWNRDCPQ
C. wingfieldii	SPCRACGEWHWREFCPL
T. mesenterica	FPCRTCGEDHWHNKCPI
