# Supplementary material for: Genetic determinants that permit growth without the core septin Cdc12 in Cryptococcus neoformans
Source: G3 (Bethesda). 2026 Aug 29;16(11):jkag240. doi: 10.1093/g3journal/jkag240 (PMC13732481; doi:10.1093/g3journal/jkag240)
Supplement: Supplementary file 1 [file jkag240_supplementary_data.zip › Supplementary_Table_1_G3-2026-407083.docx]

**Supplementary Table 1. Primers used in the study**

| **Primer name** | **Gene target** | **Primer sequence** |
| --- | --- | --- |
| **PRS 56** | **CDC12 (CNAG_01740)** | **CGT TGACTTCATTGACGACC** |
| **PRS 57** | **CDC12 (CNAG_01740)** | **GTTGTGGAGATCTTCTGGTG** |
| **PRS 58** | **CDC3 (CNAG_05925)** | **CGAGACAAAGCTCTATCAGC** |
| **PRS 59** | **CDC3 (CNAG_05925)** | **GCAATGATGGGGATAAGGTT** |
| **PRS 60** | **CNAG_05305** | **GACTCCGTCCTATCTACCTC** |
| **PRS 61** | **CNAG_05305** | **GAATGTCTCGTTTGGACCAA** |
